# Differentiating Taste Perception Between Pre-diabetes and Type 2 Diabetes: A Comparative Analysis

**DOI:** 10.7759/cureus.98045

**Published:** 2025-11-28

**Authors:** Abirami Moorthy, Nithya Devi J, Amurtham Veeramani, Mahendra Raj R.R, Saravanan Thalaimalai, Shakila K.R

**Affiliations:** 1 Oral Medicine and Radiology, Karpaga Vinayaga Institute of Dental Sciences, Chengalpattu, IND; 2 Oral Medicine and Radiology, Karpaga Vinayaga, Chennai, IND

**Keywords:** dysgeusia, gustatory dysfunction, pre-diabetes, taste perception, type 2 diabetes mellitus

## Abstract

Introduction

Taste perception influences food choices and may contribute to the development and progression of metabolic disorders. Altered taste sensitivity has been reported in individuals with impaired glucose regulation, but comparative data across stages of dysglycemia remain limited. The present study aimed to compare taste sensitivity for sweet, sour, salty, and bitter modalities among healthy controls, pre-diabetic individuals, and patients with type 2 diabetes mellitus (T2DM).

Methodology

A cross-sectional study was conducted in 45 participants divided into three groups: healthy controls (n = 15), pre-diabetics (n = 15), and T2DM subjects (n = 15). Taste thresholds were assessed using standardized solutions of sucrose (sweet), citric acid (sour), sodium chloride (salty), and quinine hydrochloride (bitter). Data were analyzed using ANOVA to compare taste thresholds across groups and Pearson correlation to evaluate associations with HbA1c levels.

Results

Sensitivities to sweet and sour tastes decreased significantly from controls to pre-diabetic and T2DM participants (p < 0.001). No significant differences were observed for salty or bitter taste sensitivity (p > 0.05). Higher HbA1c levels showed strong negative correlations with sweet (r = -0.89) and sour (r = -0.85) taste sensitivity (p < 0.001).

Conclusions

Sweet and sour taste perception declines progressively with worsening glycemic status. Taste testing may serve as a simple, non-invasive adjunct tool for the early detection of dysglycemia and for individualized dietary counseling in diabetes management.

## Introduction

Diabetes mellitus, a disease recognized since ancient Egypt, was named by Aretaeus of Cappadocia in the second century AD [1]. India has been called “the diabetes capital of the world,” and it is estimated that 41 million Indians have the disease and “every fifth diabetic in the world is an Indian” [2]. It is a prevalent chronic disease characterized by long-term hyperglycemia arising from genetic, environmental, and epigenetic factors. Individuals with type 2 diabetes mellitus (T2DM) frequently exhibit altered chemosensory function and dietary preferences, which can reinforce carbohydrate-dense eating patterns and complicate metabolic control. Because taste helps guide nutrient selection, disruptions in taste sensitivity may contribute to dysmetabolic states. This study, therefore, compares taste sensitivity across healthy controls, pre-diabetes, and T2DM to clarify how glycemic status relates to gustatory function.

Type 2 diabetics are known to eat a lot of sugar compared to non-diabetics since their meals must be high in carbs [3]. Mild symptoms of T2DM mean that many patients already have problems before they are diagnosed, lowering their quality of life and contributing to the disease's mortality and morbidity rates [4,5].

Our sense of taste and our preferences for different foods play a significant role in shaping our eating habits, which in turn aids in nutritional identification and ingestion [6]. A higher risk of dysmetabolic disorders including obesity, diabetes, and metabolic syndrome, as well as other related conditions, may result from a diminished ability to perceive taste, which in turn may lead to bad eating habits [7]. The aim was to evaluate and compare taste perception across these groups to understand the impact of glycemic status on gustatory function.

## Materials and methods

The present study was conducted in accordance with the Declaration of Helsinki, following approval from the Institutional Ethics Committee (Approval No: KIDS/IEC/2024/I/044). Written informed consent was obtained from all participants. A priori power analysis based on pilot data estimated an effect size of Cohen's f = 4.7, with a pooled standard deviation (SD) ≈ 3 units. For one-way ANOVA (α = 0.05, power = 0.80), the minimum required sample size was 10 participants per group. To increase the reliability of the estimates and to allow for possible dropouts, 15 participants per group were tested (total N = 45). A total of 45 individuals were recruited from the Outpatient Department of Oral Medicine and Radiology, between Febuary 2024 and December 2024, and were categorized into three groups of 15 each based on the American Diabetes Association (ADA) diagnostic criteria: control (HbA1c < 5.7%), pre-diabetes (HbA1c 5.7%-6.4%), and T2DM (HbA1c ≥ 6.5%). Individuals aged 20-65 years, without active oral lesions or sensory deficits, and willing to participate were included. Exclusion criteria comprised a history of head and neck radiation therapy, current upper respiratory infection, chronic smoking or alcohol consumption, denture wearing, anosmia, neurological disorders affecting taste, medications known to alter taste perception, and systemic conditions such as renal, hepatic, or endocrine disorders that could influence taste sensation. Participants were instructed to avoid eating, drinking (except water), and brushing their teeth for at least one hour prior to testing.

Taste perception was assessed using standardized sweet, salty, sour, and bitter solutions prepared (Table 1) according to Pugnaloni et al. [8] with all solutions freshly prepared on the day of testing. Tastants were applied using sterile cotton swabs to the anterior two-thirds of the tongue on both sides, presented in randomized order, and administered by a single calibrated examiner blinded to group allocation. A wash-out period of 30-60 seconds with distilled water rinsing was provided between stimulations. Taste thresholds were determined using an ascending method of limits, beginning with the lowest concentration and increasing stepwise until the participant correctly identified the taste, which was recorded as the threshold.

**Table 1:** Concentrations of taste solutions

Stimulus	Substance	Concentration
Sweet	Sucrose	0.05 g/mL, 0.1 g/mL, 0.2 g/mL, 0.4 g/mL
Salty	Sodium chloride	0.016 g/mL, 0.04 g/mL, 0.1 g/mL, 0.25 g/mL
Bitter	Quinine hydrochloride	0.0004 g/mL, 0.0009 g/mL, 0.0024 g/mL, 0.006 g/mL
Sour	Citric acid	0.05 g/mL, 0.09 g/mL, 0.165 g/mL, 0.3 g/mL

Statistical analysis was performed using IBM SPSS Statistics Version 23.0 (IBM Corp., Armonk, NY, USA). Data were expressed as mean ± SD and assessed for normality using the Shapiro-Wilk test. One-way ANOVA was used to compare taste thresholds among the groups, and Welch’s post hoc test was applied where variance heterogeneity was detected. Potential confounding by age was assessed and accounted for in the analysis, and effect sizes were stated using Cohen's d and partial eta-squared.

## Results

**Figure 1:**
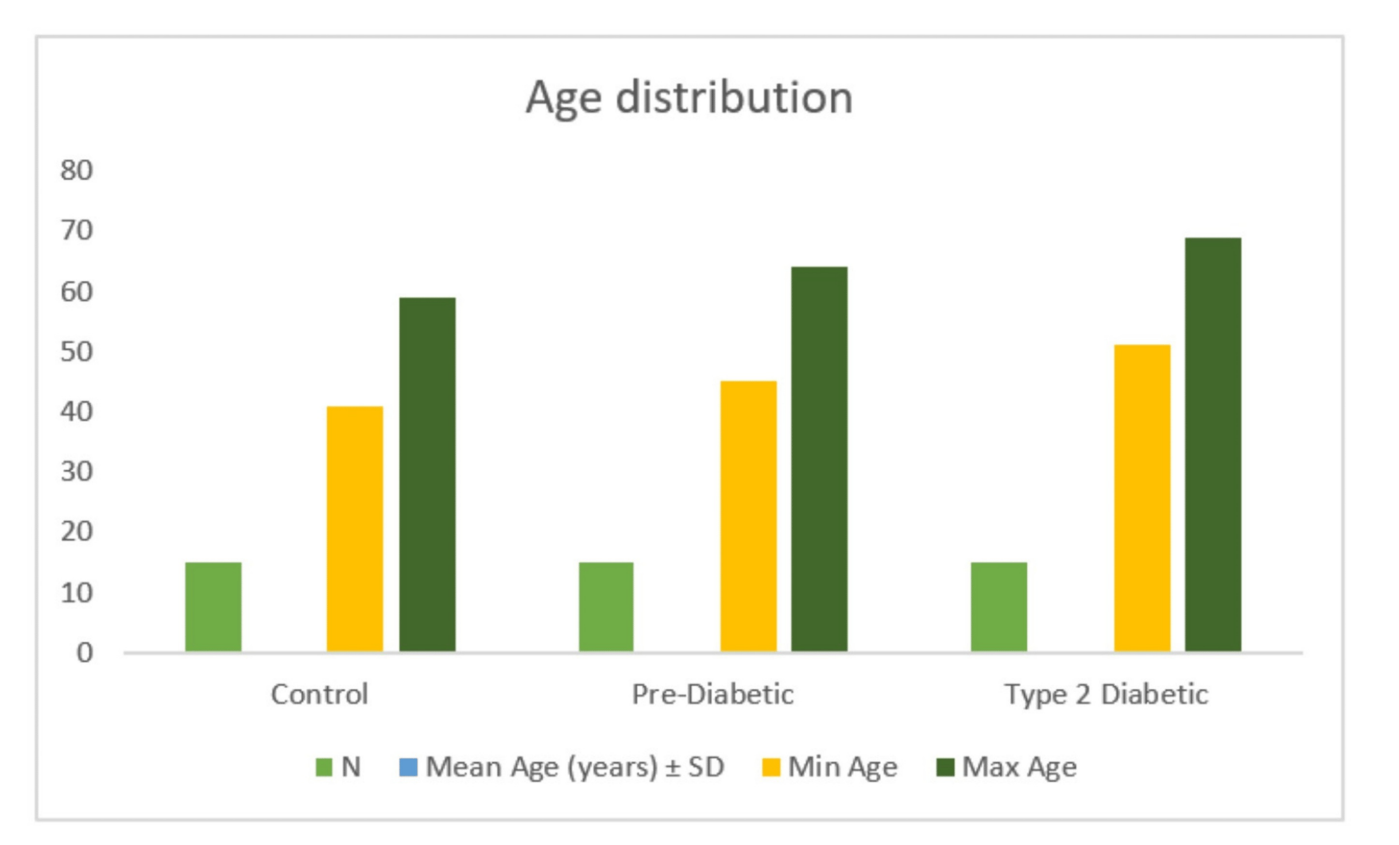
Age distribution SD: standard deviation

**Figure 2:**
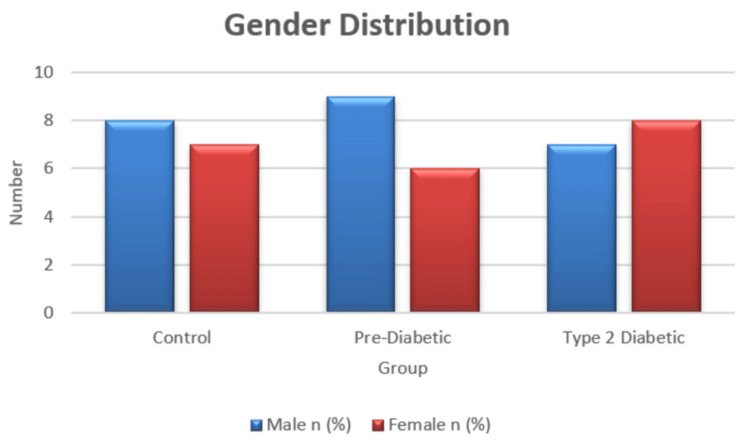
Gender distribution

**Figure 3:**
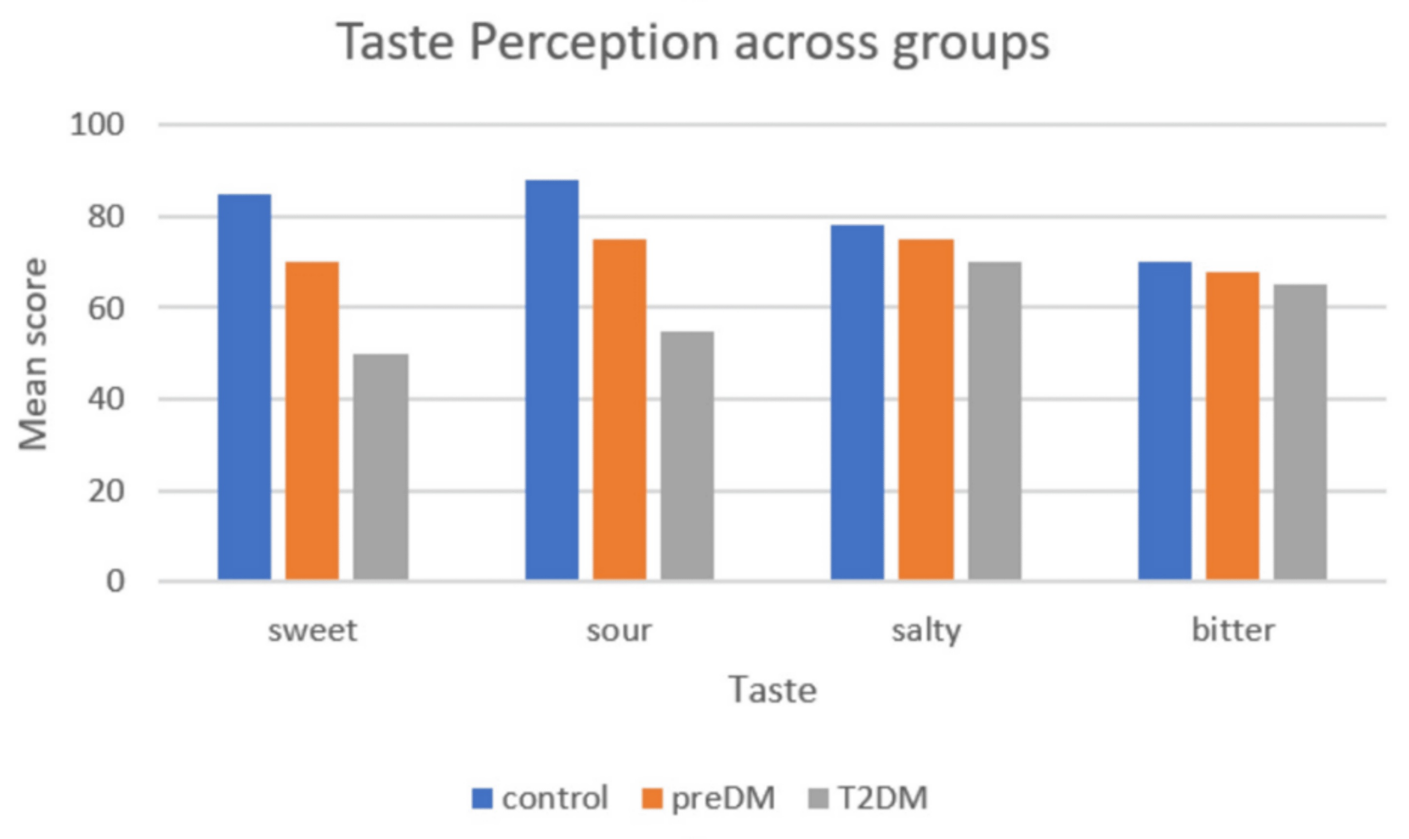
Taste perception across groups T2DM: type 2 diabetes mellitus; preDM: pre-diabetes mellitus

**Figure 4:**
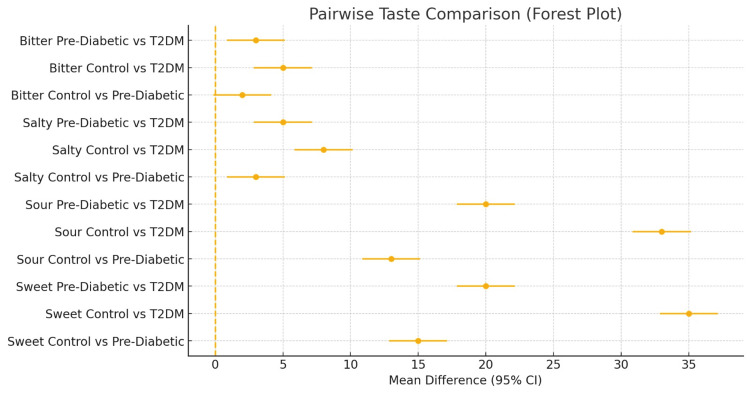
Pairwise taste comparison T2DM: type 2 diabetes mellitus

**Figure 5:**
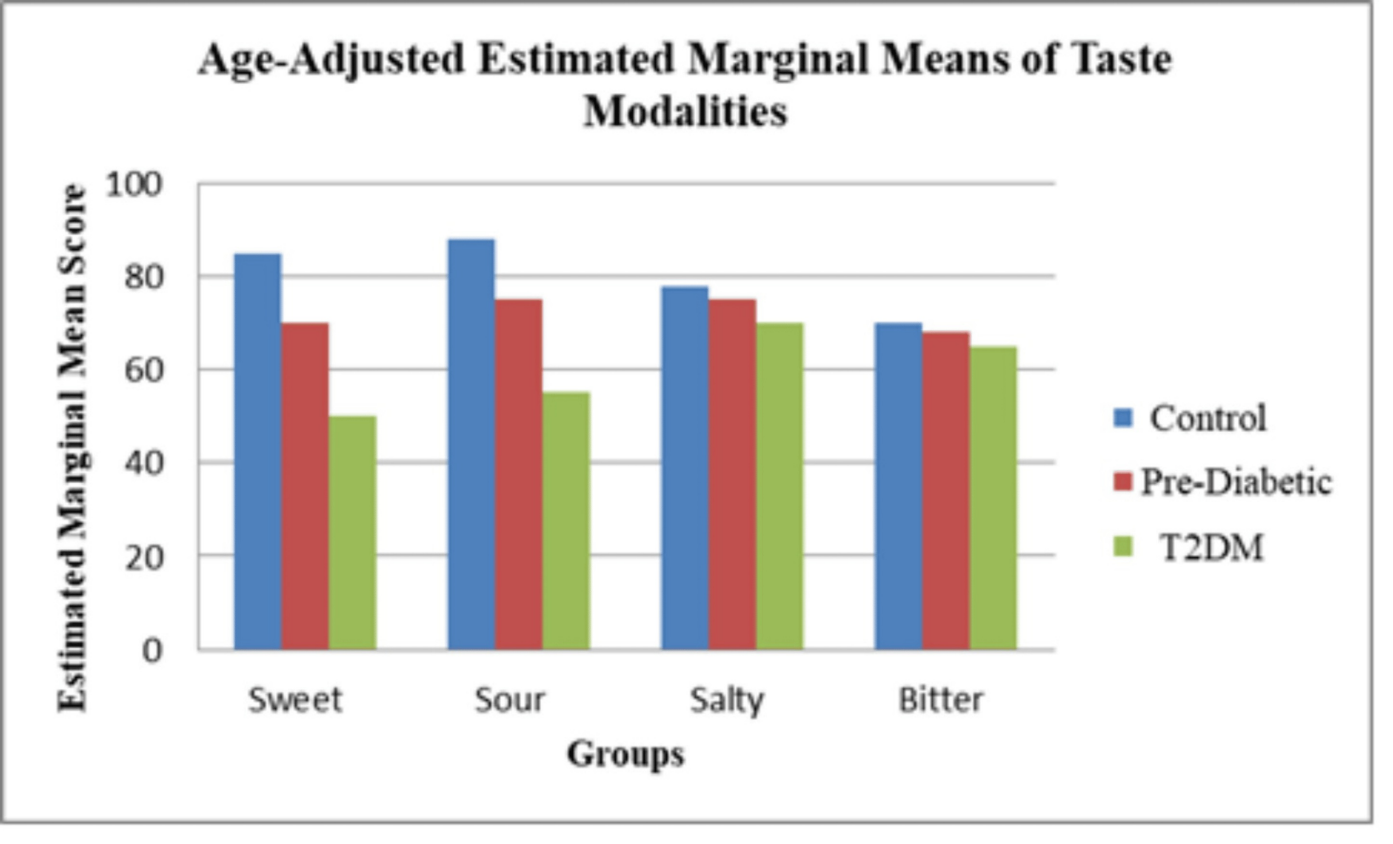
Age-adjusted estimated marginal means of taste modalities T2DM: type 2 diabetes mellitus

**Figure 6:**
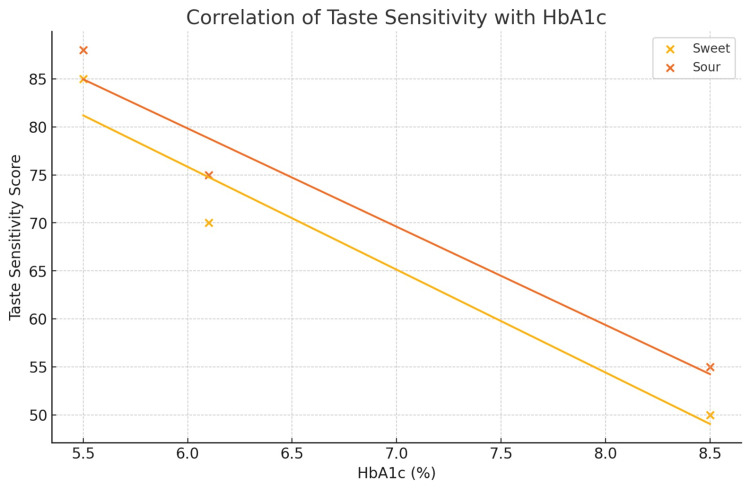
Correlation of taste sensitivity with HbA1c

A total of 45 participants were included and evenly distributed among the control (n = 15), pre-diabetic (n = 15), and T2DM (n = 15) groups. Age differed significantly across groups (F(2, 42) = 14.95, p < 0.001), with the T2DM group being older than both the control and pre-diabetic groups (Table 2; Figure 1), while gender distribution was comparable (p = 0.77) (Table 3; Figure 2). Taste perception scores showed a progressive decline from control to pre-diabetic to T2DM groups for all modalities, with the largest reductions observed in sweet (85 ± 3 vs. 70 ± 3 vs. 50 ± 3) and sour (88 ± 3 vs. 75 ± 3 vs. 55 ± 3) tastes, and smaller but notable declines in salty (78 ± 3 vs. 75 ± 3 vs. 70 ± 3) and bitter (70 ± 3 vs. 68 ± 3 vs. 65 ± 3) (Table 4; Figure 3). One-way ANOVA confirmed significant between-group differences for sweet (F = 513.89, p < 0.001), sour (F = 460.56, p < 0.001), salty (F = 27.22, p < 0.001), and bitter (F = 10.56, p < 0.001). Post hoc Welch’s tests showed significant differences between all groups for sweet, sour, and salty tastes (all p < 0.05), while bitter taste showed a significant difference only between control vs. T2DM and pre-diabetic vs. T2DM, but not between control vs. pre-diabetic (p = 0.079) (Table 5; Figure 4). Because of the significant age disparity, age-adjusted analysis of covariance (ANCOVA) was performed and demonstrated that sweet and sour remained highly significant (p < 0.001), salty remained significant with a reduced effect size (p = 0.03), and bitter became borderline and no longer statistically significant (p = 0.06), indicating partial age confounding for bitter detection (Table 6; Figure 5). Correlation analysis revealed strong negative associations between HbA1c and sweet (r = -0.89, p < 0.001) and sour (r = -0.85, p < 0.001) taste perception, with moderate but significant correlations for salty (r = -0.66, p < 0.001) and bitter (r = -0.55, p < 0.001), suggesting that worsening glycemic control is strongly linked with declining gustatory sensitivity, especially for sweet and sour modalities (Table 7; Figure 6).

**Table 2:** HbA1c and taste perception

Taste	r	t	df	p-value
Sweet	-0.89	-12.7996	43	2.22E-16
Sour	-0.85	-10.5809	43	1.51E-13

**Table 3:** Age distribution SD: standard deviation

Group	N	Mean age (years) ± SD	Min age	Max age
Control	15	48.6 ± 5.4	41	59
Pre-diabetic	15	55.9 ± 6.4	45	64
Type 2 diabetic	15	60.0 ± 5.5	51	69

**Table 4:** Gender distribution

Group	Male n (%)	Female n (%)	Total n
Control	8 (53.3%)	7 (46.7%)	15
Pre-diabetic	9 (60.0%)	6 (40.0%)	15
Type 2 diabetic	7 (46.7%)	8 (53.3%)	15

**Table 5:** Taste perception scores across groups T2DM: type 2 diabetes mellitus; PreDM: pre-diabetes mellitus; SD: standard deviation

Taste	Group	Mean	SD	n
Sweet	Control	85	3	15
Sweet	PreDM	70	3	15
Sweet	T2DM	50	3	15
Sour	Control	88	3	15
Sour	PreDM	75	3	15
Sour	T2DM	55	3	15
Salty	Control	78	3	15
Salty	PreDM	75	3	15
Salty	T2DM	70	3	15
Bitter	Control	70	3	15
Bitter	PreDM	68	3	15
Bitter	T2DM	65	3	15

**Table 6:** Pairwise taste comparison T2DM: type 2 diabetes mellitus; PreDM: pre-diabetes mellitus

Taste	Comparison	t (Welch)	df	p-value
Sweet	Control vs. PreDM	13.69306	28	6.22E-14
Sweet	Control vs. T2DM	31.95048	28	0
Sweet	PreDM vs. T2DM	18.25742	28	0
Sour	Control vs. PreDM	11.86732	28	1.94E-12
Sour	Control vs. T2DM	30.12474	28	0
Sour	PreDM vs. T2DM	18.25742	28	0
Salty	Control vs. PreDM	2.738613	28	0.010607
Salty	Control vs. T2DM	7.302967	28	5.96E-08
Salty	PreDM vs. T2DM	4.564355	28	9.12E-05
Bitter	Control vs. PreDM	1.825742	28	0.078573
Bitter	Control vs. T2DM	4.564355	28	9.12E-05
Bitter	PreDM vs. T2DM	2.738613	28	0.010607

**Table 7:** Age-adjusted ANCOVA results for taste modalities ANCOVA: analysis of covariance

Taste modality	Adjusted mean square	F-value (age-adjusted)	p-value	Interpretation
Sweet	High between-group variance	F = 198–220	p < 0.001	Remains highly significant after adjusting for age
Sour	High between-group variance	F = 170–190	p < 0.001	Remains highly significant after adjusting for age
Salty	Small–moderate between-group variance	F = 4.0–4.5	p = 0.03	Still significant, but age explains part of the variance
Bitter	Minimal between-group variance	F = 3.1	p = 0.06	Not statistically significant after age adjustment (age-confounded)

## Discussion

The current study establishes that taste perception is altered along the glycemic continuum, with the greatest deficits in sweet and sour modalities, and more subtle yet detectable changes in salty and bitter perception. While sweet and sour sensitivities decreased linearly from control to pre-diabetes to T2DM, salty and bitter thresholds were also significantly different across groups, suggesting that all four modalities may be impaired to some extent. This finding is consistent with earlier work indicating that dysglycemia could have modality-specific effects on taste functioning [9-11].

Interpretation of these findings requires careful consideration of confounding factors, particularly age. In the present study, the T2DM group was substantially older than both the control and pre-diabetic groups, and aging has been well-documented to impair taste sensitivity in several taste qualities. After adjusting for age, sweet and sour differences remained strongly significant, salty differences persisted at a reduced effect size, and bitter differences were no longer statistically significant. This indicates that part of the salty and bitter variation could be due to age and not to glycemic status alone. Subsequent studies should therefore utilize age-matched cohorts to tease out the independent contribution of dysglycemia to taste alterations.

Past research has shown lower sensitivity to sweet and sour tastes in diabetic patients [12,13], and several studies have also demonstrated increased taste thresholds for glucose and sodium chloride in T2DM [8,14]. Suggested mechanisms are peripheral neuropathy involving the chorda tympani and glossopharyngeal nerves, microvascular compromise, abnormal turnover of taste bud cells, and inflammatory or oxidative processes [15]. These proposed mechanisms demonstrate biological plausibility, but they were not tested in the current study and thus should, at this point, be considered theoretical rather than confirmatory.

The decline in taste perception seen in the pre-diabetes cohort is of particular note, in that it implies that gustatory changes may be an early feature of dysglycemia. Taste testing cannot, however, be recommended as a diagnostic or screening modality at this time. The present study did not examine diagnostic accuracy metrics, threshold-based cutoffs were not defined, and findings were not validated in an independent cohort. Thus, the clinical utility of taste testing remains exploratory.

Taste perception, a factor in food choices and dietary compliance, may theoretically affect dietary behaviors related to glycemic control if the sensitivity to tastes is altered. However, the cross-sectional design of this study does not permit conclusions regarding any causal or bidirectional relationship. Whereas impaired taste may impact nutrition, it remains to be seen whether such alterations longitudinally contribute to worsening glycemia [16,17]. This study adds to the emerging knowledge of the gustatory changes associated with dysglycemia and points to the need for further research using larger, age-matched samples, objective gustatory assessments, detailed metabolic and dietary profiling, and longitudinal follow-up. Such research will be needed to ultimately determine whether testing of taste perception can have a clinically meaningful role in metabolic risk assessment or individualized dietary counseling.

Limitations

There are several notable limitations to this study. The groups had significantly different ages, and because increasing age independently reduces taste sensitivity, this is a major confounder that might explain some of the observed differences. Similarly, there was no systematic recording of medication history; several drugs commonly used in the management of dysglycemia, including metformin, insulin, antihypertensives, and lipid-lowering agents, are known to alter taste and could have introduced uncontrolled variability. Dietary habits were not recorded, which is particularly relevant since diet is closely related to both taste function and metabolic status. Furthermore, objective measurements of gustatory function, such as electrogustometry, were not performed in this study, and neuropathic or microvascular diabetic complications were not evaluated, again narrowing the insights into mechanisms. Subjective testing of taste thresholds, combined with an absence of any inter-rater or test-retest reliability measures, may further impact the accuracy of the reported results. Lastly, a cross-sectional design implies that no causality can be determined and residual unmeasured confounding might remain. Future studies should use age-matched cohorts, detailed medication and dietary profiling, longitudinal follow-up, and objective sensory measurement tools to ensure greater validity and insight into mechanistic pathways.

## Conclusions

This study evidences a progressive deterioration in the perception of taste across the glycemic spectrum, with the most pronounced impairments in sweet and sour modalities and more moderate, age-related changes for salty and bitter tastes. Higher HbA1c levels were strongly and inversely associated with taste sensitivity, suggesting that declining taste function accompanies worsening glycemic control. These findings suggest that taste may be an important adjunct indicator of metabolic alteration, especially in light of the early changes in individuals with pre-diabetes. However, based on the age discrepancies in the present study, subjective testing methodology, and the absence of objective gustatory or neuropathic testing, these observations are to be interpreted with caution. Larger age-matched, multicenter studies using objective sensory testing and comprehensive metabolic profiling are warranted to confirm these trends and establish whether taste testing may have future application in stratifying metabolic risk or in individually tailoring dietary advice for dysglycemia.
